# Mindfulness-based cognitive therapy as a treatment for chronic depression: A preliminary study

**DOI:** 10.1016/j.brat.2009.01.019

**Published:** 2009-05

**Authors:** Thorsten Barnhofer, Catherine Crane, Emily Hargus, Myanthi Amarasinghe, Rosie Winder, J. Mark G. Williams

**Affiliations:** Department of Psychiatry, University of Oxford, Warneford Hospital, Oxford OX3 7JX, UK

**Keywords:** Depression, Chronic, Recurrence, Mindfulness, Meditation, MBCT

## Abstract

This pilot study investigated the effectiveness of Mindfulness-Based Cognitive Therapy (MBCT), a treatment combining mindfulness meditation and interventions taken from cognitive therapy, in patients suffering from chronic-recurrent depression. Currently symptomatic patients with at least three previous episodes of depression and a history of suicidal ideation were randomly allocated to receive either MBCT delivered in addition to treatment-as-usual (TAU; *N* = 14 completers) or TAU alone (*N* = 14 completers). Depressive symptoms and diagnostic status were assessed before and after treatment phase. Self-reported symptoms of depression decreased from severe to mild levels in the MBCT group while there was no significant change in the TAU group. Similarly, numbers of patients meeting full criteria for depression decreased significantly more in the MBCT group than in the TAU group. Results are consistent with previous uncontrolled studies. Although based on a small sample and, therefore, limited in their generalizability, they provide further preliminary evidence that MBCT can be used to successfully reduce current symptoms in patients suffering from a protracted course of the disorder.

## Introduction

In a significant number of those affected, Major Depression follows a protracted lifetime course with patients suffering from either full episodes or sub-syndromal levels of symptoms over considerable amounts of time (Kessing, Hansen, & Andersen, 2004; Solomon et al., 2000). While views of depression often stress its episodic nature, it has also become clear that in many cases, patients do not recover fully from episodes but continue to show residual symptoms, which themselves have been found to be an important predictor of relapse (Judd et al., 1998; Paykel et al., 1995; Pintor, Gasto, Navarro, Torres, & Fananas, 2003; Pintor, Torres, Navarro, Matrai, & Gasto, 2004). In particular, after severe episodes, sub-syndromal levels of depression are common and persistent (Kennedy, Abbott, & Paykel, 2004). In other patients, symptoms remain relatively stable at the level of full episodes over periods of more than 24 months (Mueller et al., 1996). Recent research has indicated that regardless of their presentation, i.e., whether patients continue to suffer from syndromal levels of the disorder or fluctuate between syndromal and sub-syndromal levels, chronic forms of depression are broadly homogeneous with regard to both their clinical and etiological features, while, at the same time, differing in important regards from episodic forms of the disorder (McCullough et al., 2003). For example, individuals suffering from chronic forms of depression have been found to be more likely to have a familial history of chronic depression (Klein et al., 1995), to be more likely to have suffered from early adversity (Lizardi et al., 1995), to be more likely to suffer from high levels of chronic stress (Klein, Taylor, Dickstein, & Harding, 1988; Ravindran, Griffiths, Waddell, & Anisman, 1995) and neuroticism (Hirschfeld, Klerman, Andreasen, & Clayton, 1986; Weissman, Prusoff, & Klerman, 1978), and to be more likely to suffer from co-morbid disorders, particularly personality (Garyfallos et al., 1999; Pepper et al., 1995) and anxiety disorders (Weissman, Leaf, Bruce, & Florio, 1988). Most importantly, chronic forms of depression have been found to be significantly less responsive to treatment (Thase, Reynolds, Frank, & Simons, 1994) with reports of rates of responders to single modality treatments in trials aimed at chronic depression or based on samples with highly recurrent forms of depression at about 50% (DeRubeis et al., 2005; Keller et al., 2000). There is, thus, an important need for further refinement of treatments for those who have developed a more protracted course of the disorder.

Mindfulness-Based Cognitive Therapy (MBCT; Segal, Williams, & Teasdale, 2002) is a treatment programme that was specifically designed to address latent vulnerability in depression. It combines training in mindfulness meditation and interventions from cognitive therapy for acute depression and is delivered in a group setting. The rationale of the treatment is based on findings from cognitive research on vulnerability that has linked relapse to mood-related reactivation of negative thinking patterns (Lau, Segal, & Williams, 2004; Scher, Ingram, & Segal, 2005) and maladaptive ways of responding to negative cognitions and emotions such as rumination (e.g., Watkins, 2008), thought suppression (Wenzlaff & Bates, 1998) and experiential avoidance, i.e., an unwillingness to remain in contact with one's private experiences, leading to attempts at altering experience so that it is less aversive (Hayes et al., 2004). Through the use of mindfulness meditation, participants are taught to develop their ability to recognize and disengage from maladaptive forms of negative automatic and repetitive thinking. In two randomized controlled trials (RCTs), in which previously depressed patients were followed up over a period of one year following the treatment phase, MBCT has been found to reduce risk of relapse by approximately half in patients with three or more previous episodes of depression (Ma & Teasdale, 2004; Teasdale et al., 2000). A recent study has found MBCT to be as effective in reducing relapse over a follow-up period of 15 months as maintenance therapy with antidepressants (Kuyken et al., 2008).

Each of these three previous RCTs of MBCT studied only patients who were in remission or recovery. The use of MBCT in *current* depression had been discouraged because it was unclear whether the cognitive demands of a regular meditation practice would exceed the restricted capacities of depressed patients. However, as factors specifically addressed by MBCT are likely to play an important role not only in recurrence but also in the maintenance and persistence of depression, interest in applying MBCT to a wider range of patients has recently increased. In chronically depressed patients, maladaptive thinking patterns such as rumination and experiential avoidance are likely to have acquired a habitual nature, and mental training using mindfulness meditation may hold particular promise in reversing such tendencies. In line with this, there are now a number of reports that suggest that MBCT can successfully reduce symptoms in currently depressed patients (Finucane & Mercer, 2006; Kingston, Dooley, Bates, Lawler, & Malone, 2007), with two of the studies showing effects in patients who had been found to be resistant to established forms of treatment (Eisendrath et al., 2008; Kenny & Williams, 2007). However, with the exception of the study by Kingston et al. which only included participants with residual symptoms, all of these reports are based on uncontrolled pre–post-comparisons.

The purpose of the current study was to carry out a preliminary study to investigate the effects of MBCT in patients suffering from chronic forms of depression using a randomized controlled design with blind assessments. We compared the immediate effects of MBCT delivered in addition to treatment-as-usual (TAU) to TAU alone. At this stage of knowledge and given the high vulnerability of this group, we offered MBCT to the TAU group as soon as the post-treatment assessments were complete, effectively establishing a waitlist condition. The study focused on patients with a history of suicidality in the past, a group in which cognitive vulnerability has been found to be particularly pronounced (Williams, Barnhofer, Crane, & Beck, 2005; Williams, Van der Does, Barnhofer, Crane, & Segal, 2008), thus, addressing the most severe end of the depressive spectrum. We hypothesized that participants in the MBCT condition would show significant decreases in severity of depressive symptoms, while no such changes were expected in the TAU group, and that number of responders at the end of the treatment phase would be significantly higher in the MBCT than in the TAU condition.

## Method

### Participants

The study had received full approval by the Mid and South Buckinghamshire Local Research Ethics Committee (Ref: 07/Q1607/2). Participants were recruited through local media advertisements and posters as well as referrals from local mental health practitioners. Interested individuals were screened in a telephone interview and those indicating current presence of core symptoms of depression (feeling sad or depressed, or loss of interest) and a history of chronic or recurrent depression with suicidal ideation were invited for an assessment session at the Department of Psychiatry. In this session, eligibility was assessed using the Structured Clinical Interview for DSM-IV-TR Axis I (SCID; First, Gibbon, Spitzer, & Williams, 2002) and the DSM-IV module for Borderline Personality Disorder, in order to assess presence of habitual self-harming, administered by a trained clinical research psychologist. Inclusion criteria were (a) a history of at least three previous episodes of Major Depression or Chronic Depression, i.e., a full episode of Major Depression lasting for at least two years, (b) a current diagnosis of Major Depression or presence of residual symptoms following a full episode, defined as either meeting DSM-IV criteria for only four instead of at least five symptoms of depression over the last two weeks or suffering from five or more symptoms for at least half of the days, if symptoms had not been present for most of the days over the past two weeks, (c) a history of suicidal ideation (including thoughts of methods of suicide) or suicidal behavior, (d) absence of current mania or hypomania, psychosis, obsessive-compulsive disorder, eating disorder, pervasive developmental disorder or habitual self-harming, substance abuse or dependence that would significantly interfere with the ability to engage in meditation, (e) adequate written and spoken English to complete all study measures, (f) not currently in individual or group psychotherapy and no current ongoing meditation practice, and (g) age between 18 and 65. In addition to our own assessments, we required written confirmation from the participant's GP that there was no contraindication for him or her to take part in the study.

**Fig. 1:**
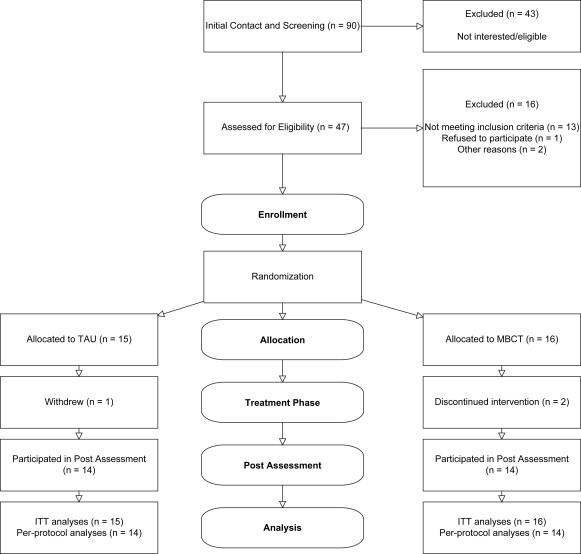
Flowchart depicting passage of participants through the trial.

A total of 90 individuals made an initial contact in response to information regarding the study, of which 43 did not participate. The main reasons for people who had made an initial contact not to participate were (a) they were not interested after hearing more about the study or had other commitments that would have interfered with participation in the study (14, 32%), (b) the research team was unable to contact them for a telephone interview after their initial contact (7, 16%), and (c) they did not meet criteria for the study (22, 53%; six current level of depression below inclusion criteria, three not suicidal in the past, four current mania or hypomania, three current eating disorder, two current psychotic symptoms, one severe habitual self-harm, three older than 65 years of age). Of the 47 participants, who were invited for an interview at the Department of Psychiatry, 13 were found not to be eligible (three current level of depression below inclusion criteria, four habitual self-harming, one pervasive developmental disorder, one mania, one hypomania, one alcohol dependence, one eating disorder, one would not have been available at time of classes). Three further participants were lost following the interview: one withdrew before randomization and before completing any measures, for one participant consent from their GP could not be obtained, and with one participant who, during the interview, had displayed significant deficits in orientation, memory and attention, the origin of which was currently unknown but seemed likely to be neurological, it was mutually decided that participation in the classes was too demanding and, therefore, contraindicated. This left a sample of 31 participants to be randomized into the study. Of those who had been randomized, three participants, two from the MBCT group and one from the TAU group, dropped out before the post-treatment phase assessments. Full data sets were, therefore, available for *N* = 14 participants allocated to MBCT and *N* = 14 to TAU. The flow of the participants through the trial is depicted in Fig. 1.

In order to test the feasibility of MBCT for chronically depressed patients at this early stage of development, the study was restricted to only one cohort. Previous pre–post-comparisons of MBCT in currently depressed patients had produced effects sizes of *d* = 1.00 (Eisendrath et al., 2008, Kenny & Williams, 2007). In order to find effects of a similar size with power of 0.80 at an *α*-level of 0.05, we would have had to recruit 16 participants into each group. However, given that effect sizes in a control group design are likely to be smaller, the current study was underpowered.

Characteristics of the two groups are summarized in Table 1 along with the results of *t*-tests for continuous variables and *χ*^2^-tests for binary variables. The two groups were comparable in their sociodemographic characteristics including age, gender distribution, their relationship status, years of education and employment. Most participants were middle-aged, about two thirds were women and about half of the participants were married or co-habiting.

Overall, the sample was characterized by a protracted course of the disorder with early onset and high rates of chronicity and recurrence. There were significantly more individuals who were classified as suffering from a chronic full episode in the TAU group. However, the two groups were comparable with regard to both the average duration of the last episode of depression, when residual symptoms were taken as part of an ongoing episode, i.e., length of time until full recovery, as well as the overall duration of the disorder, i.e., number of months that individuals had suffered from the disorder. About two thirds of both groups were in full episode at baseline while the remaining third suffered from symptoms at sub-threshold levels after having been in a full episode. None of the participants, therefore, were in remission, so none would have been entered into any of the three previous RCTs of MBCT (Kuyken et al., 2008; Ma & Teasdale, 2004; Teasdale et al., 2000).

About 80% of participants had previously received treatment with antidepressant medication and about 60% were taking antidepressants when the trial started: four participants tricyclic antidepressants, seven selective serotonin reuptake inhibitors, three serotonine–norepinephrine reuptake inhibitors, two tetracyclic antidepressants, and one an atypical antipsychotic as their main medication. The two groups did not differ significantly with regard to the numbers of participants who were currently taking antidepressant medication.

More than two thirds of the participants had previously received some form of psychotherapy or counseling over at least five sessions and more than half of them had previously received CBT with rates of those who had previously received psychological treatment comparable in the two groups.

### Procedure

All participants provided written informed consent prior to the start of the research assessments. They took part in two sessions both before and after the treatment phase at the Department of Psychiatry, Warneford Hospital. Pre-assessments were conducted within 2 ½ months of the start of the treatment phase, from the start of recruitment in mid February until end of April 2007 (mean number of days between first assessment and start of treatment phase: TAU: *M* = 49.00, SD = 17.27; MBCT: *M* = 40.79, SD = 15.17, *F* (1, 26) = 1.78, *p* = 0.19), and post-assessments took place within 1 month after the end of the treatment phase, from the beginning to end of July 2007 (mean number of days from end of treatment phase to first post-assessment: TAU: *M* = 12.00, SD = 7.61; MBCT: *M* = 11.64, SD = 6.24; *F* (1, 26) = 0.01, *p* = 0.89). During the first of the two sessions, the structured clinical interview was conducted and participants completed several self-report questionnaires as well as a number of cognitive tasks. Assessors did not have access to any information about the participants that related to their group status and participants were explicitly instructed not to give any hints regarding their group membership during the post-assessment interview. The second session was used to assess EEG parameters of brain functioning, the results of which are not reported here. Participants were randomly allocated to either MBCT or TAU after eligibility was ascertained in the first assessment session pre treatment. Randomization was conducted using sealed envelopes prepared by a statistician from outside of the research team using a computer generated randomization sequence (blocks of 4, no stratification) that remained concealed until assignment to the groups. Participants were informed about assignment by the research administrator coordinating the study while interviewers and assessors remained blind.

### Measures

#### Structured Clinical Interview for DSM-IV-TR (SCID; First et al., 2002)

The Research Version of the Structured Clinical Interview for DSM-IV-TR Axis I Disorders and the Borderline Personality Disorder Module of the Structured Clinical Interview for DSM-IV Axis II were used to assess current and past diagnostic status at entry into the study. In order to facilitate tracking of more complex courses of depression, the interview was supplemented with a visual grid that served as a timeline on which the beginning and end of previous episodes of depression were marked. Post-treatment assessment interviews used the mood disorders module of the SCID only and focused on the time since the first interview in order to assess any change in diagnostic status.

#### Beck Depression Inventory-II (BDI-II; Beck, Steer, & Brown, 1996)

Self-reports on the BDI-II were used to measure severity of current depressive symptoms pre- and post-treatment. The BDI-II contains 21 statements, assessing symptoms over the preceding two weeks. Beck, Steer, and Brown report excellent internal consistency in both patient and student samples.

#### Beck Scale for Suicide Ideation (BSS; Beck & Steer, 1991)

The BSS was used to measure presence and severity of suicidal ideation. The self-report questionnaire comprises 21 groups of statements including a screening section that contains five items specifically designed to identify suicidal ideation in active or passive form. Individuals whose answers in the screening section indicate suicidal ideation are asked to answer 14 further items assessing the extent of their wish to die. Two further items ask for information about previous suicide attempts. Analyses reported here use a sum score of the screening items in order to compare presence of suicidal ideation before and after treatment in both groups. Internal consistency of the screening items in our sample was *α* = 0.87 at pre-assessment and *α* = 0.90 at post-assessment.

### Treatment

Participants of both groups were encouraged to continue any current medication and to attend appointments with their mental health practitioners or other services over the treatment phase as they would have done otherwise. MBCT and TAU participants were entered into the study only if they did not currently receive any form of individual psychotherapy and were asked not to start one during the time of the study. TAU participants were, additionally, asked not to start a regular meditation practice during the time of the study.

Before the start of the treatment phase participants in the MBCT group were invited for a pre-class interview with the therapist to prepare them for the course while participants in the TAU group met with the research coordinator to discuss further procedures until the start of their treatment after the waiting period. The MBCT treatment followed the manual by Segal et al. (2002) with some minor alterations to address suicidality and presence of acute symptoms, i.e., introduction of crisis plans and cognitive components addressing suicidal cognitions and hopelessness. Handouts and protocol sheets for home practice were taken from the manual without major alteration. Participants in the MBCT condition met in a group of, initially, 16 patients for eight weekly classes of 2 h duration in June and July 2007. In addition to the classes, they were asked to engage in homework including regular meditation or mindful movement practice and various other related exercises for about an hour per day for six days a week. The classes were led by a fully-qualified CBT therapist who had trained in Mindfulness-Based Stress Reduction through an internship at the Center for Mindfulness in Medicine, Health Care and Society at the University of Massachusetts, Worcester, Medical Center (TB), and who delivered the treatment under regular supervision of JMGW, one of the developers of the programme, who monitored adherence to the treatment protocol.

An overview of services and treatments participants used as part of their TAU is given in Table 2. Over the course of the treatment phase, seven of the participants in the TAU group changed their medication: two participants who had not been on antidepressants at the start of the study resumed taking antidepressant medication, two participants changed their medication to include a new, additional antidepressant, one increased the dosage of the current medication, and two lowered the dosage of their medication. In the MBCT group, two participants changed medication over the course of the treatment phase, one went off their medication and one increased the dosage of their medication. Differences in number of participants in the two groups who changed medication were marginally significant, *Fisher's Exact Test*, *p* = 0.052. However, as this difference was due mostly to increased use of antidepressants in the TAU group, any effect on outcome measures would have worked against our hypotheses and change in medication was, therefore, not used as a covariate in outcome analyses. Groups were comparable with regard to use of services in all other categories assessed. Psychological treatments were used by six of the participants in the TAU group (one attended a single follow-up appointment with their GP, one a single appointment with a mental health nurse, one participant received two sessions of counseling, and two had a number of appointments with their psychiatrist that included CBT-type interventions) and four of the participants in the MBCT group (three participants had appointments with their counselor, one participant one appointment, one participant two appointments, and one participant four appointments; one other participant in this group started participating in therapeutic group meetings in a religious context towards the end of the treatment phase). Seven of the participants in the TAU group and eight of the participants in the MBCT group had appointments with their GP related to their depressive disorder, one or more times, during the treatment phase, and in both groups, four of the participants had one or more visits from a psychiatric nurse that was related to their depressive disorder. Six of the participants in each group reported looking into some form of self-help approach during the treatment phase, reading books about depression and mental health or searching for related information on the internet.

### Outcome measures

Analyses of outcome focussed on changes in level of depressive symptoms as assessed by self-reports on the BDI-II and changes in diagnostic status as assessed by SCID interview conducted by blind assessors. Treatment response was defined by combination of two criteria: a 50% reduction in BDI-II scores from pre- to post-treatment together with a post-treatment BDI-II score of 13 or below, which is defined as the threshold level for mild depressive symptoms.

## Results

### Treatment acceptance

Participants who completed the MBCT treatment attended an average of *M* = 6.14 (SD = 1.51) classes. Homework records indicated that they had practiced meditation or yoga on an average of 4.98 days out of the six days they had been asked to practice between sessions for about an hour per day. There were no adverse events that were deemed to be related to the treatment. One participant in the MBCT group contacted the therapist during a suicidal crisis and after crisis intervention was referred to their psychiatrist. This patient continued to participate in the classes and assessments.

### Changes in severity of depressive symptoms

Intention-to-treat analyses were conducted first, including all participants who were randomized into the study. For this purpose, the BDI-II scores of those three participants who withdrew from the study after randomization were carried forward, from pre- to post-assessment. Carrying forward the baseline scores of those who withdraw is a conservative approach of data imputation since it assumes no change in these participants. Resulting average severity of symptoms as measured with BDI-II was *M* = 29.36 (SD = 9.66) at pre-assessment and *M* = 17.62 (SD = 10.94) at post-assessment in the MBCT group, and *M* = 31.32 (SD = 10.79) at pre-assessment and *M* = 28.86 (SD = 12.97) at post-assessment in the TAU group. A 2 (time: pre versus post) × 2 (group: MBCT versus TAU) repeated measures ANOVA of BDI-II scores with time as within- and group as between-subjects factor yielded a significant main effect of time, *F* (1, 29) = 13.42, *p* = 0.001, partial *η*^2^ = 0.32, that was qualified by a significant time by treatment interaction, *F* (1, 29) = 5.74, *p* = 0.02, partial *η*^2^ = 0.17, *M*_*I*_ _−_ _*J*_ = 11.74, SE = 2.69, *p* < 0.001, partial *η*^2^ = 0.39, 95% CI: lower bound = −3.23, upper bound = 8.15, in the MBCT group and *M*_*I*_ _−_ _*J*_ = 2.45, SE = 2.78, *p* = 0.38, partial *η*^2^ = 0.02, 95% CI: lower bound = 6.23, upper bound = 17.25, in the TAU group. This interaction remained significant when duration of the last episode was taken into account as a covariate, *F* (1, 28) = 5.01, *p* = 0.03.

Second, we analysed the per-protocol sample. This showed average BDI-II scores of *M* = 30.35 (SD = 9.9) at pre-assessment and *M* = 16. 92 (SD = 11.53) at post-assessment in the MBCT group, and *M* = 32.06 (SD = 10.80) at pre-assessment and *M* = 29.42 (SD = 13.27) at post-assessment in the TAU group. The 2 (time: pre versus post) × 2 (group: MBCT versus TAU) repeated measures ANOVA of BDI-II scores with time as within- and group as between-subjects factor yielded a significant main effect, *F* (1, 26) = 15.37, *p* = 0.001, partial *η*^2^ = 0.37, qualified by a significant group by time interaction, *F* (1, 26) = 6.9, *p* = 0.014, partial *η*^2^ = 0.21. Bonferroni-corrected follow-up tests showed the change in symptoms in the MBCT group to be significant, *M*_*I*_ _−_ _*J*_ = −13.42, SE = 2.89, *p* < 0.001, partial *η*^2^ = 0.45, 95% CI: lower bound = −7.47, upper bound = −19.37, while change in the TAU group was non-significant *M*_*I*_ _−_ _*J*_ = 2.63, SE = 2.89, *p* = 0.37, partial *η*^2^ = 0.03, 95% CI: lower bound = −3.38, upper bound = 8.58. Again, results remained statistically significant when analyses were re-run with length of current episode in months as a covariate, *F* (1, 25) = 6.16, *p* = 0.02 for the time by treatment interaction.

### Rates of response

Intention-to-treat analyses of treatment responses included those participants who withdrew from the study following randomization as non-responders. Using the above described combined criterion, a reduction in BDI-II score of at least 50% and a post-treatment BDI-II score of 13 or below, 6 out of the 16 participants randomized to MBCT were classed as responders, yielding a response rate of 37%. In contrast, in the TAU group, only 1 out of the 15 participants randomized into this group showed decreases in symptoms that would have fulfilled these criteria, yielding a rate of 6%. A *χ*^*2*^-test showed this difference to be significant, *χ*^*2*^ (*N* = 31) = 4.21, *p* = 0.04.

Analyses based on the per-protocol sample yielded response rates of 43% (6 out of 14 participants) in the MBCT group and 7% (1 out of 14) in the TAU group. As in the intention-to-treat analysis, a *χ*^*2*^-test showed this difference to be significant, *χ*^*2*^ (*N* = 28) = 4.76, *p* = 0.02.

### Change in diagnostic status

We also analysed changes in numbers of participants who met criteria for a full episode of Major Depression as assessed by SCID interview conducted by assessors blind to treatment allocation. For these analyses, it is important to keep in mind that, as some of the participants did not meet full criteria for an episode of Major Depression at entry into the study, diagnostic status can reflect positive change, i.e., changes from diagnostic to non-diagnostic status, only in a subgroup of participants. Table 3 provides an overview of numbers of participants meeting full criteria for MDD at pre- and post-assessment. As all three of the participants who withdrew over the course of the study did not meet full criteria for diagnostic status at entry, the below analyses are based entirely on participants from the per-protocol sample. Of those who had met criteria for a full episode of depression at entry into the study, 7 out of 10 participants (70%) in the MBCT group did not meet criteria for a full episode of Major Depression at post-assessment, while this was the case for only 2 out of 11 participants (18%) in the TAU group, Fisher's Exact Test *p* = 0.03 (Table 3).

### Change in suicidal ideation

Changes in suicidal ideation as assessed by self-reports on the screening items of the BSS were analysed using 2 (time: pre versus post) × 2 (group: MBCT versus TAU) repeated measures ANOVAs. Neither analyses in the intent-to-treat nor the per-protocol sample yielded significant effects (intent-to-treat sample: time × group interaction, *F* (1, 29) = 0.16, *p* = 0.50; per-protocol sample: time × group interaction, *F* (1, 26) = 0.52, *p* = 0.47). In the per-protocol sample, average BSS screening scores at pre- and post-assessment, respectively, were *M* = 2.21 (SD = 2.45) and *M* = 1.14 (SD = 1.79) in the MBCT group and *M* = 2.78 (SD = 2.08) and *M* = 2.42 (SD = 2.53) in the TAU group.

## Discussion

The aim of this study was to conduct a preliminary randomized controlled trial to investigate whether MBCT can successfully reduce symptoms of depression in currently symptomatic patients who suffer from a protracted course of the disorder and in whom spontaneous recovery is relatively unlikely to occur. Previous RCTs had excluded such patients on the grounds that MBCT might be inappropriate unless patients were in remission or recovery; though there had been promising results from uncontrolled trials (Eisendrath et al., 2008; Finucane & Mercer, 2006; Kenny & Williams, 2007). Consistent with these studies, our results showed that treatment with MBCT significantly reduced self-reported symptoms of depression from severe to mild levels, while levels of depression remained unchanged in the group that received TAU only. Numbers of patients who met criteria for Major Depression decreased in the MBCT group but remained in the TAU group.

While the current study extends previous research by using a randomized controlled design and blind assessments, there are a number of limitations that need to be taken into account. First of all, and most importantly, this study is based on only a small sample of patients. Because of this, the study is potentially more vulnerable to spurious effects and generalizability of its findings is more uncertain. One potential difficulty that comes with small numbers is that randomization is more likely to fail to produce groups that are comparable in all important respects. The two groups here differed with regard to the proportion of participants classified as currently being in a full chronic episode of depression as compared to suffering from residual symptoms following a full episode. Because of this imbalance, it could be argued that part of the differential changes in symptoms found may be due to participants in the TAU group being less likely to show spontaneous recovery because of the more chronic nature of their depression. While this possibility cannot be completely ruled out, there are several points that speak against it. First, when length of current episode was used as a covariate in analyses of BDI-II changes, results remained significant. Second, previous research on chronic depression has demonstrated that, while diagnostic systems differentiate between chronic episodes of depression and recurrent depression without full inter-episode remission, these two forms share more similarities than there are differences between them in terms of clinical features and risk factors (McCullough et al., 2003). Consistent with this, the two groups in our study, although different in terms of numbers of those suffering from chronic episodes, did not differ in total length of time they had suffered from depression in their lifetime nor length of the last episode when ongoing residual symptoms were taken as part of the episode.

A second important limitation of the study is that the main findings regarding severity of depressive symptoms are based on self-reports, which are amenable to subjective biases. Ideally, these measures would have been complemented by observer-rated measures of symptom severity. However, while there was no such measure for symptom severity, the study did include structured interviews to assess diagnostic status before and after the treatment phase. Although comparisons of numbers of participants who qualified for a full diagnosis of depression were restricted because some of the participants did not meet full criteria for a diagnosis of Major Depression at pre-assessment, analyses yielded significant effects indicating stronger decreases in number of patients meeting full criteria in the MBCT group (70% no longer meeting diagnostic criteria) than in the TAU group (18%).

The current sample was characterized by a protracted course of the disorder and all of the participants had suffered from suicidal ideation in the past. Both established psychotherapeutic and pharmacological treatments have been found to fail to help a significant number of these patients. For example, in a large recent study of cognitive therapy for severe depression, where many of the patients included reported a recurrent or chronic course of the disorder, rates of response were 58% (DeRubeis et al., 2005). A large study investigating a treatment specifically designed for chronic depression, the Cognitive Behavioral-Analysis System of Psychotherapy (CBASP), found satisfactory responses after 16–20 sessions of individual therapy in 48% of patients, a rate that increased to 73% in those who received both CBASP treatment in addition to psychopharmacological treatment with the antidepressant nefazodone (Keller et al., 2000). Given the relatively low intensity of MBCT with only eight weekly sessions and the economic advantage of the treatment being delivered in groups, the rates of response found in our study can be judged as generally encouraging. Direct comparisons of rates of response between this and other studies need to take into account that the current study used a combined criterion requiring both severity levels below a certain threshold level and percentage change as compared to use of a threshold level only.

Despite the fact that, initially, the developers of MBCT had cautioned against its use in currently depressed patients, rates of attendance at classes and the data from participants' records of homework practice suggest that the treatment is both acceptable and manageable for this group. These data were in line with the clinical impressions from the classes. Because of their current symptoms, we had expected that patients would encounter more negative content during their meditations and one particular concern had been that, especially in the early stages of the treatment, participants might get easily overwhelmed by this negative content and find it more difficult to use the meditation techniques to their advantage. In cases where this seemed to be a problem, we offered participants the opportunity to choose their home practice more flexibly, and, when needed, to use practices that provided particularly tangible anchors for their attention such as sensations of the body during yoga stretches or the sensations of the breath during sitting meditations. Another variation offered to participants was to experiment, in sitting meditations, with opening their eyes at times when they found content to be overwhelming and to use this technique to approach difficult content in a stepwise manner. Clinical impressions suggested that only a small number of patients required and made use of these variations of the standard practice.

While results showed significant decreases in severity of depressive symptoms, we did not find significant changes in suicidal ideation. In part, this may be due to lack of power for detecting such differences as a large number of patients entered the study with relatively low levels of suicidal ideation. Taken as they are, though, these findings suggest the need to address suicidal thinking in even more detail. Possible additions could, for example, include a psychoeductional component on the impact of suicidal imagery and the immediacy with which such imagery can occur and impact. Recent research has suggested that suicidal patients, when at their most despairing, often suffer from intrusive mental images of suicide and that the frequency and subjective reality of such images is significantly related to severity of suicidal ideation (Holmes, Crane, Fennell, & Williams, 2007). Yet, available treatments so far have not included a specific focus on this aspect of psychopathology.

Research on the effects of meditation has been growing over the recent years and studies suggest that regular meditation practice can produce lasting effects on attention, executive functioning (Lutz, Slagter, Dunne, & Davidson, 2008; Slagter et al., 2007; Tang et al., 2007) and emotion regulation (Nielsen & Kaszniak, 2006). Systematic training of these functions through meditation may be particularly helpful in patients suffering from chronic depression where vulnerability processes such as rumination are likely to have acquired a habitual and automatic character and are more likely to occur when cognitive control is undermined. This small-scale randomized controlled trial comparing MBCT to TAU represents a logical next step in testing the application of MBCT in chronic depression, following positive reports from clinical audits. Altogether, the findings provide preliminary evidence suggesting that using mindfulness meditation in the treatment of chronic or recurrent depression is feasible and may provide a valuable addition to already established interventions. Further research into the effects of mindfulness meditation will help to tailor the MBCT program more specifically to the needs of this group. The focus here was on immediate effects and future studies need not only replicate the current findings but should also use more extensive follow-ups given the high risk of relapse in the histories of these patients as well as active treatment comparisons in order to control for unspecific effects of the MBCT training.

## Figures and Tables

**Table 1:** Sample characteristics of the MBCT (*N* = 14) and TAU (*N* = 14) groups.

	MBCT	TAU	Test-statistic	*p*
Age *M* (SD)	42.07 (11.34)	41.79 (9.52)	*t* (26) = 0.07	ns
Gender (female/male)	10/4	9/5	*X*^2^ = 0.16	ns
Married/co-habiting *n* (%)	7 (50)	7 (50)	*χ*^2^ (*N* = 28) = 0.00	ns

Years of education *M* (SD)	16.38 (3.04)	15.21 (3.19)	*t* (25) = 0.97	ns
Employed *n* (%)	13 (92)	11 (78)	*χ*^2^ (*N* = 28) = 1.16	ns

Current MDD (full episode/residual symptoms)	10/4	11/3	*χ*^2^ (*N* = 28) = 0.19	ns
Age of Onset *M* (SD)	20.57 (7.64)	23.21 (11.68)	*t* (26) = 0.71	ns
Number of episodes *Mde* (range)	4 (1–50)	3 (1–6)	Mann-Whitney *U* = 58	ns

Number of episodes *M* (SD)	7.76 (12.9)	3.07 (1.89)	*t* (24) = 1.29	ns
Chronic Depression *n* (%)^a^	7 (50)	12 (85)	*χ*^2^ (*N* = 28) = 4.09	0.04

Duration of last episode in months *M* (SD)	87.92 (149.80)	51.07 (41.20)	*t* (26) = 0.88	ns
Total duration in months *M* (SD)	116.50 (141.32)	87.23 (43.23)	*t* (23) = 0.71	ns
History of manic or hypomanic episode *n* (%)	1 (7)	1 (7)	*χ*^2^ (*N* = 28) = 0.00	ns
Co-morbid anxiety disorder *n* (%)	4 (28)	6 (42)	*χ*^2^ (*N* = 28) = 0.62	ns
Co-morbid somatoform disorder *n* (%)	2 (14)	0 (0)	*χ*^2^ (*N* = 28) = 2.15	ns
Co-morbid axis I disorder (one or more) *n* (*%*)	6 (42)	6 (42)	*χ*^2^ (*N* = 28) = 0.00	ns
Previous suicide attempt *n* (*%*)	4 (28)	4 (28)	*χ*^2^ (*N* = 28) = 0.00	ns
Previous psychotherapy or counseling *n* (%)^b^	10 (71)	11 (79)	*χ*^2^ (*N* = 28) = 0.19	ns
Previous CBT *n* (*%*)	7 (50)	8 (57)	*χ*^2^ (*N* = 28) = 0.14	ns

Past treatment with antidepressant medication *n* (%)	11 (79)	12 (86)	*χ*^2^ (*N* = 28) = 0.24	ns
Current treatment with antidepressant medication *n* (%)	9 (64)	8 (57)	*χ*^2^ (*N* = 28) = 0.15	ns

a *Note*. Participants with episodes of MDD lasting at least two years or recurrent depression without full inter-episode recovery over an equivalent period of time.

b More than 5 sessions.

**Table 2:** Number (and percentages) of participants in the MBCT (*N* = 14 completers) and TAU (*N* = 14 completers) receiving particular services and treatments during the treatment phase.

	MBCT	TAU	Fisher's Exact Test *p*
Changed antidepressant medication	2 (14)	7 (50)	0.052
Received psychological intervention	4 (28)	6 (42)	ns
Visited GP regarding depression	8 (57)	7 (50)	ns
Received visit by psychiatric nurse	4 (28)	4 (28)	ns
Use of self-help (books etc.)	6 (42)	6 (42)	ns

**Table 3:** Numbers of Participants meeting DSM-IV criteria for Major Depression at pre- and post-assessment in the MBCT (*N* = 14 completers) and TAU groups (*N* = 14 completers).

			Post-assessment	
			MDD	No MDD
Pre-Assessment	MBCT	MDD	3	7
		No MDD	1	3
	TAU	MDD	9	2
		No MDD	0	3

*Note*. MDD = major depressive disorder.
